# Transcriptional determinants of tolerogenic and immunogenic states during dendritic cell maturation

**DOI:** 10.1083/jcb.201512012

**Published:** 2017-03-06

**Authors:** Bryan Vander Lugt, Jeremy Riddell, Aly A. Khan, Jason A. Hackney, Justin Lesch, Jason DeVoss, Matthew T. Weirauch, Harinder Singh, Ira Mellman

**Affiliations:** 1 Department of Cancer Immunology, Genentech, South San Francisco, CA 94080; 2 Department of Bioinformatics and Computational Biology, Genentech, South San Francisco, CA 94080; 3 Department Translational Immunology, Genentech, South San Francisco, CA 94080; 4 Center for Autoimmune Genomics and Etiology, Divisions of Biomedical Informatics and Developmental Biology, Cincinnati Children’s Hospital Medical Center, Cincinnati, OH 45229; 5 Center for Systems Immunology, Division of Immunobiology, Cincinnati Children’s Hospital Medical Center, Cincinnati, OH 45229; 6 Institute for Systems and Genomics Biology, The University of Chicago, Chicago, IL 60637; 7 Department of Human Genetics, The University of Chicago, Chicago, IL 60637

## Abstract

Dendritic cells (DCs) promote either tolerogenic or immunogenic T cell responses, the latter upon sensing microbes. Using an in vitro system, we analyzed transcriptional determinants that enable mature DCs to direct these opposing T cell outcomes. In the absence of microbial products, the transcription factor interferon regulatory factor 4 (IRF4) promotes regulatory T cell (T_reg_) generation by enhancing expression of genes required for antigen presentation along with those for T cell tolerance. IRF4-deficient DCs were impaired for T_reg_ generation in vivo. When exposed to microbial stimuli, DCs activated nuclear factor (NF)-κB, which induced expression of a proinflammatory cytokine module that, along with the antigen presentation module, promoted the generation of effector T cells. NF-κB was, however, dispensable for T_reg_ development. Chromatin profiling revealed transcriptional motifs associated with the divergent DC programs. Thus, DCs modulate their ability to prime tolerogenic or immunogenic T cells by expressing a core antigen presentation module that is overlaid by distinctive regulatory modules to promote either tolerance or immunity.

## Abstract (precis)

Dendritic cells promote either immunosuppressive or immunogenic T cell responses, but the transcriptional and epigenetic programs regulating these functions are unclear. Vander Lugt et al. dissect the distinct programs underlying the immunogenic and tolerogenic mature states of dendritic cells in vitro.

## Introduction

Dendritic cells (DCs) not only present peptide antigens to T cells but also deliver important secondary signals that shape ensuing immune responses (Mellman and Steinman, 2001). Pathogen- or inflammation-associated products license DCs to promote the differentiation of T cells into diverse effector states (T_eff_) that are tailored to effectively counter the infecting agent (Joffre et al., 2009). Such danger cues trigger dramatic alterations in DC organization and function, including enhanced antigen processing and surface display of peptide major histocompatibility complex class II (MHCII) complexes, induced expression of costimulatory molecules, and production of inflammatory cytokines necessary for T_eff_ polarization (Trombetta and Mellman, 2005). Danger-induced terminal differentiation of DCs, referred to as DC maturation, is thought to coordinately regulate these transformations and enhance DCs’ ability to prime T_eff_ generation (Joffre et al., 2009).

In the steady state (the absence of infection or danger), DCs foster immune tolerance to self and innocuous environmental antigens (Steinman et al., 2003). This is accomplished in part by promoting the differentiation of naive T cells into immunosuppressive regulatory T cells (T_reg_). Migratory DCs (MigDCs) constitutively present self or innocuous antigens during homeostasis (Scheinecker et al., 2002) and are particularly adept at promoting T_reg_ differentiation (Idoyaga et al., 2013). Intriguingly, steady-state MigDCs appear phenotypically mature, in that they express high levels of MHCII and costimulatory molecules but do not provoke autoimmune responses (Ruedl et al., 2000). In addition, steady-state MigDC maturation occurs normally in germ-free mice and mice lacking signaling adaptors that transmit microbial cues (Wilson et al., 2008; Baratin et al., 2015). Such observations suggest that DCs can undergo maturation independently of the pathogen-derived or proinflammatory signals required for immunogenicity. Furthermore, the findings imply that, depending on the signals received during maturation, DCs can manifest distinctive states with tolerogenic or immunogenic potential.

The transcriptional and epigenetic programs that underlie tolerogenic and immunogenic states of DCs have yet to be elucidated (Dalod et al., 2014). Because DCs associated with tolerance in the steady state can exhibit a mature phenotype, we reasoned that a danger-independent “core” maturation program may exist that transcriptionally regulates antigen presentation/costimulatory functions and enables DCs to engage naive T cells. If so, then tolerogenic or immunogenic signals should activate distinct transcriptional determinants that regulate the tolerogenic versus immunogenic potential of a mature DC. It seems likely that such transcriptional programs would represent components of regulatory modules that are overlaid on the core DC maturation module. Although the existence of tolerogenic and immunogenic DCs is well established from in vivo studies, we know little regarding the underlying genomic regulatory mechanisms because of inadequate utilization of a model experimental system that enables analysis of the divergent DC maturation programs. We therefore investigated our hypotheses using a DC maturation model system that enables precise control and perturbation of DC differentiation under either tolerogenic or immunogenic conditions. In so doing, we not only provide experimental support for our hypothesis but reveal shared as well as distinctive transcriptional determinants that orchestrate the programming of the prototypic and divergent DC functional states.

## Results

### Coupling of steady-state DC maturation with tolerogenic programming

We recently described use of a bone marrow–derived dendritic cell (BMDC) culture system to analyze the functions of transcription factors interferon regulatory factor 4 (IRF4) and IRF8 in regulating DC maturation as well as MHCII antigen presentation and priming of helper T cell (Th) responses. Using this system, we demonstrated that both transcription factors (TFs) promoted DC maturation, but IRF4 preferentially enhanced expression of genes involved in MHCII antigen processing and presentation, thereby enabling more efficient priming of Th responses. This experimental system, which makes use of granulocyte/macrophage colony-stimulating factor (GM-CSF) and interleukin (IL)-4, has been extensively used to characterize the unique cell biological properties that distinguish dendritic cells from macrophages (Mellman and Steinman, 2001). Many of the discoveries made with this in vitro system have been confirmed in vivo with particular DC subsets. GM-CSF has been shown to promote the differentiation of hematopoietic progenitors into cells resembling macrophages and dendritic cells, as revealed by the expression of CD115 or CD135, respectively (Helft et al., 2015). A substantial fraction of cells in GM-CSF cultures undergo further maturation and exhibit increased expression of surface MHCII and costimulatory molecule(Inaba et al., 1992). Notably, the inclusion of IL-4 with GM-CSF inhibits the differentiation of CD115^hi^ macrophage-like cells (Jansen et al., 1989; Helft et al., 2015), thereby biasing toward the generation of DCs.

To further clarify the relationship between the cells generated in vitro using GM-CSF and IL-4 and tissue-resident DCs, particularly given a recent study (Helft et al., 2015), we undertook genome-wide expression profiling to comprehensively analyze the molecular profiles of GM-CSF + IL-4–derived cells with bona fide DCs. We sorted pure populations of MHCII^hi^ mature and MHCII^lo^ immature GM-CSF and IL-4 BMDCs and compared their genomic expression profiles with those of BMDCs generated using GM-CSF alone (Helft et al., 2015) and those of macrophage and DC subsets in the Immunological Genome Consortium (ImmGen) database (Heng et al., 2008). The various genome-wide expression datasets were transformed to enable comparison across distinct microarray platforms (Fig. S1 A). The analysis revealed that MHCII^hi^ and MHCII^lo^ populations from GM-CSF and IL-4 cultures have strongly correlated gene expression profiles that were quite distinct from the GM-CSF–alone populations (Fig. S1 B). Importantly, comparison of genome expression profiles of MHCII^hi^ and MHCII^lo^ GM-CSF and IL-4 BMDCs to those of macrophage and DC subsets in the ImmGen database revealed that the GM-CSF + IL-4–generated cells were more similar to the DC subsets (Fig. S1 C). In this regard, it is noteworthy that MHCII^hi^ BMDCs generated with GM-CSF + IL-4 up-regulate a set of genes that significantly overlap with those whose expression is restricted to steady-state MigDC populations (Vander Lugt et al., 2014). Recent studies have demonstrated that GM-CSF is a critical signal for the homeostatic differentiation of migratory, but not lymphoid tissue–resident, DC populations (Greter et al., 2012; Mortha et al., 2014). Thus, taking into account earlier cell and molecular biological studies with the GM-CSF and IL-4 BMDCs as well as our comparative genome-wide expression analyses, the data strongly support the utility of this in vitro model system for analyzing molecular determinants and gene regulatory networks orchestrating DC maturation and their tolerogenic or immunogenic programming.

**Figure 1.**
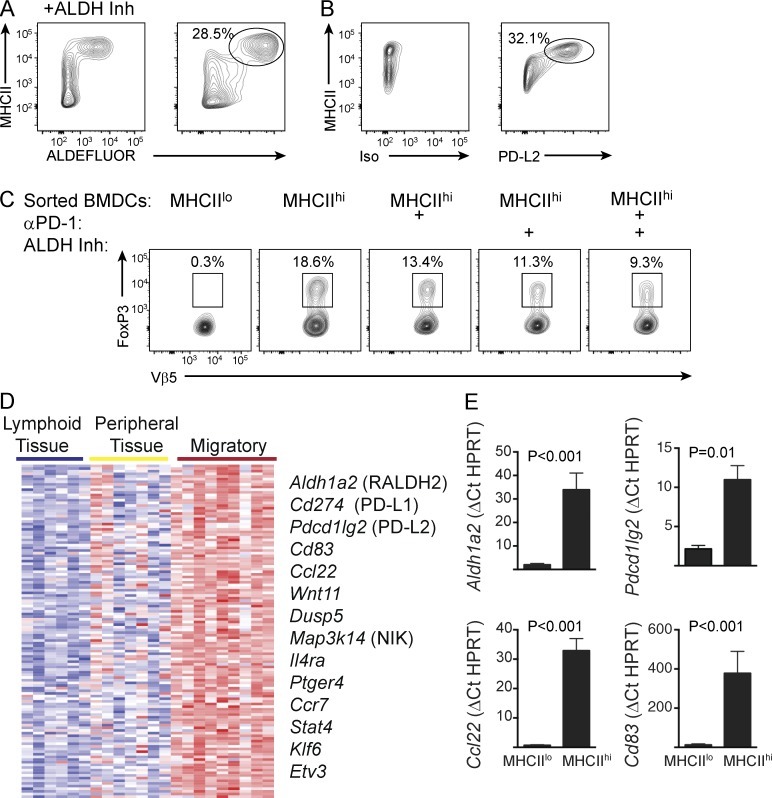
**Regulatory signals are induced with steady-state DC maturation.** (A) ALDH activity in unstimulated MHCII^hi^ BMDCs was determined by flow cytometry after administration of fluorescently labeled ALDH substrate (ALDEFLUOR) in the presence or absence of ALDH inhibitor (Inh). Data are representative of three independent experiments. (B) BMDCs were analyzed by flow cytometry as in A for PD-L2 expression or staining with isotype-matched control antibody. Data are representative of at least five independent experiments. (C) Unstimulated BMDCs were loaded with ovalbumin before isolation of MHCII^lo^ and MHCII^hi^ populations. Purified DCs were cultured with naive OT-II T cells for 5 d with or without PD-1 blocking antibody or an ALDH inhibitor. Vβ5^+^ T cells were subsequently analyzed by flow cytometry for FoxP3 expression. Numbers indicate percentage within the adjacent gate. Data are representative of at least three experiments. (D) Microarray gene expression profiles from DC populations compiled in the ImmGen database were analyzed to identify a module of 127 genes whose expression significantly correlated with *Aldh1a2*. Heatmap represents mean expression of genes (rows) from two to three replicates of select lymphoid tissue resident, peripheral tissue resident, or MigDC populations (columns). Key genes of biological interest are annotated on the right. (E) MHCII^lo^ and MHCII^hi^ populations were purified from unstimulated BMDC cultures, and expression of the indicated genes was determined by Taqman RT-PCR. Bars represent mean of three independent experiments ± SEM. Statistical significance was assessed by paired two-tailed *t* test.

The enhanced ability of MigDCs to promote T_reg_ differentiation may reflect the expression of retinaldehyde dehydrogenase (RALDH), a key enzyme for retinoic acid production (Guilliams et al., 2010). We confirmed high ALDH activity selectively among MigDCs ex vivo by flow cytometric analysis, as previously reported (Fig. S2 A; Guilliams et al., 2010). Furthermore, we demonstrated restricted expression of PD-L2 in the same DC populations, a signal that has also been proposed to regulate DC induction of peripheral T_regs_ (Zhang et al., 2006; Fukaya et al., 2010; Fig. S2 B). We therefore examined the expression of these tolerogenic components in BMDCs generated with GM-CSF and IL-4 and found that both ALDH activity and PD-L2 expression were induced upon maturation (Fig. 1, A and B). Thus, based on genome-wide profiling as well as expression of ALDH activity and PD-L2, the BMDC system recapitulates expression of functionally relevant regulatory components selectively associated with MigDC maturation and tolerogenic programming.

To directly test the acquisition of tolerogenic potential, we purified ovalbumin-loaded MHCII^lo^ immature or MHCII^hi^ mature populations from unstimulated BMDCs and co-cultured them with naive ovalbumin-specific OT-II T cells. The mature DCs, in contrast to their immature counterparts, efficiently promoted T_reg_ differentiation, as assessed by FoxP3 expression (Fig. 1 C). These results strongly suggest that immature DCs are not optimal for priming tolerogenic responses, as is often assumed because of their low-level expression of costimulatory molecules such as CD80 and CD86. Inclusion of the ALDH inhibitor to block retinoic acid production or a blocking antibody to PD-1 to inhibit PD-L2–mediated signaling impaired the generation of FoxP3-expressing T_regs_. Combined inhibition of the two tolerogenic signaling components in BMDCs resulted in a further reduction in T_reg_ differentiation. Thus, DC maturation in this in vitro system is coupled to the acquisition of tolerogenic potential in the absence of danger signals.

We next used a bioinformatic approach to more thoroughly examine the molecular profiles of steady-state mature BMDCs with a focus on genes that are specifically expressed in MigDCs and regulate their migratory and tolerogenic potentials. Using gene expression profiles of various DC populations compiled in the ImmGen database, we identified a module of 127 genes whose expression was significantly correlated with *Aldh1a2*, the gene encoding the RALDH2 enzyme critical for ALDH activity in DCs (Fig. 1 D). Intriguingly, many genes within this set encode proteins with established roles in immune tolerance, such as PD-L1, PD-L2, CD83, and CCL22 (Zhang et al., 2006; Sather et al., 2007; Sun et al., 2007; Fukaya et al., 2010; Bates et al., 2015). We verified by RT-PCR that select genes within this set were up-regulated in MHCII^hi^ mature BMDCs (Fig. 1 E). Collectively with our earlier findings (Vander Lugt et al., 2014), these results provide evidence for our hypothesis that a core program of DC maturation involving enhanced antigen presentation and expression of costimulatory molecules is juxtaposed with a distinctive molecular program that directs tolerogenic T cell responses in the absence of danger signals.

### IRF4 coordinates DC maturation with tolerogenic programming

The concurrent up-regulation of genes encoding secondary signals for T_reg_ differentiation together with those required for MHCII antigen presentation raised the possibility that there may be sharing of some regulatory determinants to coordinate these programs. We considered the TF IRF4 as such a shared determinant, because of previous findings establishing its role in regulating DC maturation and efficient MHCII antigen presentation (Tamura et al., 2005; Vander Lugt et al., 2014) as well as in controlling distinct types of immunogenic programming (Gao et al., 2013; Schlitzer et al., 2013; Williams et al., 2013). We reasoned that in the absence of danger signals, IRF4 could function in programming immunosuppressive potential in DCs.

**Figure 2.**
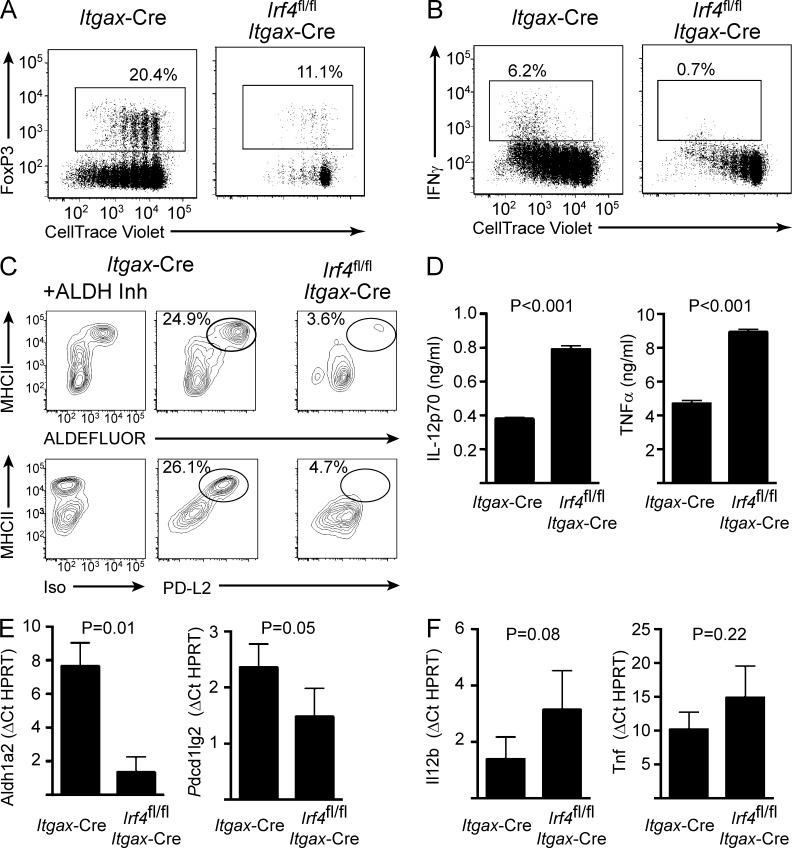
**IRF4 regulates tolerogenic maturation but not immunogenic signals.** BMDCs were generated from *Itgax*-Cre or *Irf4*^fl/fl^ *Itgax*-Cre mice. (A) Unstimulated BMDCs were loaded with ovalbumin before co-culture with CellTrace Violet–labeled naive OT-II T cells for 5 d. T cells were subsequently analyzed by flow cytometry for dye dilution and expression of FoxP3. Numbers indicate percentage within the adjacent gate. Data are representative of three independent experiments. (B) BMDCs were generated as in A, loaded with ovalbumin, and LPS-stimulated for 6 h before co-culture with CellTrace Violet–labeled naive OT-II T cells for 5 d. T cells were subsequently analyzed by flow cytometry for dye dilution and expression of IFNγ. Numbers indicate percentage within the adjacent gate. Data are representative of three independent experiments. (C) ALDH activity in unstimulated BMDCs was determined by flow cytometry after administration of fluorescently labeled ALDH substrate (ALDEFLUOR) in the presence or absence of ALDH inhibitor (Inh). BMDCs were similarly analyzed by flow cytometry for PD-L2 expression or staining with isotype-matched control antibody. (D) Concentrations of the indicated cytokines in supernatants of BMDCs stimulated as in B were determined by Luminex. Bars indicate mean of three independent experiments ± SEM. (E and F) Expression of the indicated genes was assayed in unstimulated (E) or LPS-stimulated (F) BMDCs by Taqman RT-PCR. Bars indicate mean ± SEM. Statistical significance was assessed by two-tailed *t* test.

To test our hypothesis, we generated BMDCs from *Irf4*^fl/fl^ *Itgax*-Cre mice, which enables conditional deletion of the *Irf4* gene in CD11c^+^ DCs (Vander Lugt et al., 2014). IRF4-deficient BMDCs showed a diminished capacity in promoting T_reg_ generation in vitro (Fig. 2 A). As expected, they were also impaired in their ability to prime T_eff_ cells (Fig. 2 B). These results are consistent with those in Fig. 1 C and a model invoking efficient MHCII antigen presentation as a necessary step in eliciting both T_eff_ and T_reg_ differentiation. To determine whether IRF4 specifically regulates tolerogenic programming, independent of its functions in controlling MHCII antigen presentation, we analyzed RALDH activity and PD-L2 expression in IRF4-deficient BMDCs. Expression of both components was severely reduced and was correlated with failure to up-regulate MHCII expression (Fig. 2 C). Diminished PD-L2 expression in IRF4-deficient BMDCs has also been noted by Gao et al. (2013). Consistent with the protein analysis, transcripts for *Aldh1a2* (encodes a RALDH isoform) and *Pdcd1lg2* (encodes PD-L2) were substantially reduced in *Irf4*^fl/fl^ *Itgax*-Cre DCs compared with control DCs (Fig. 2 D). In contrast, expression of the proinflammatory cytokines IL-12 and TNF, and their genes, was elevated in IRF4-deficient DCs (Fig. 2, E and F).

**Figure 3.**
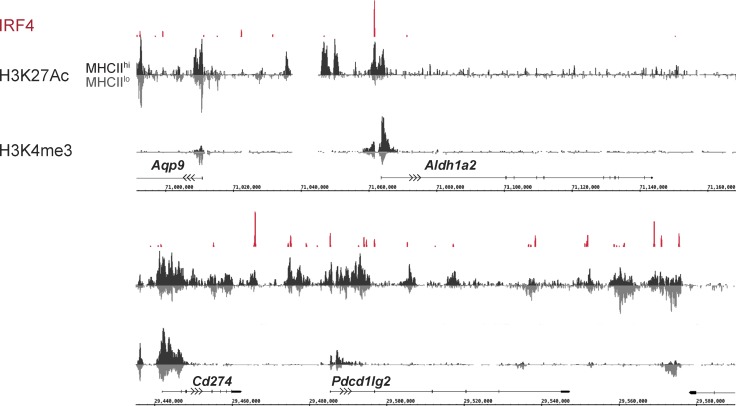
**IRF4 directly regulates DC immunosuppressive programming.** ChIPseq analysis for histone H3K27 acetylation and H3K4 trimethylation was performed on MHCII^hi^ (black) and MHCII^lo^ (gray) populations purified from unstimulated BMDCs. MHCII^lo^ track is inverted and juxtaposed with MHCII^hi^ track to facilitate comparison of signals. Our previously reported ChIPseq analysis for IRF4 binding in total CD11c^+^ BMDCs is displayed in red. Data are representative of two independent experiments.

The defect in the expression of the *Aldh1a2* and *Pdcd1lg2* genes could reflect an indirect consequence of the requirement for IRF4 in DC maturation. Therefore, we used chromatin immunoprecipitation combined with massively parallel DNA sequencing (ChIPseq) analysis to determine whether IRF4 targets these genes and directly regulates their expression. ChIPseq revealed prominent IRF4 binding peaks in the promoter regions of both genes (Fig. 3). IRF4 therefore directly regulates the expression not only of key genes in the MHCII antigen presentation and migration pathways (Vander Lugt et al., 2014), but also those encoding tolerogenic components. Thus, IRF4 appears to represent a shared regulatory determinant that couples efficient MHCII antigen presentation with tolerogenic programming during steady-state DC maturation.

### IRF4-deficient DCs are impaired for T_reg_ generation in vivo

**Figure 4.**
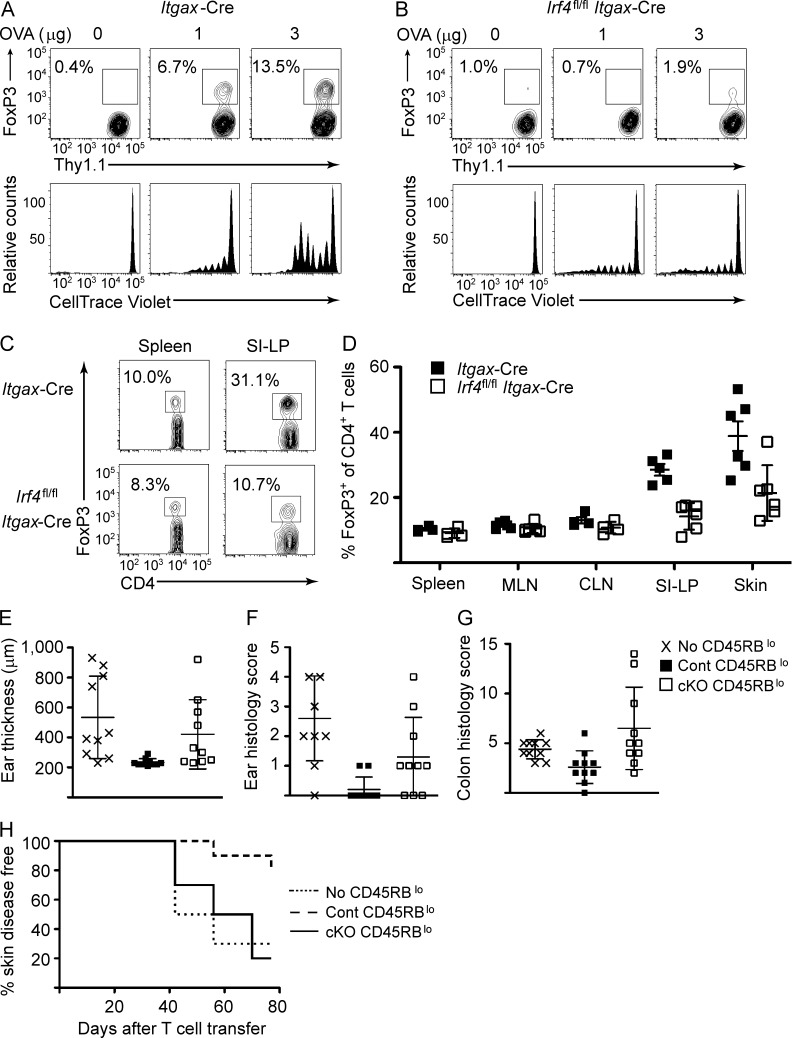
**Impaired peripheral tolerance in *Itgax*-Cre *Irf4*^fl/fl^ mice.** (A and B) Purified, CellTrace Violet–labeled Thy1.1^+^ OT-II T cells were transferred into *Itgax*-Cre or Itgax-Cre *Irf4*^fl/fl^ mice. Recipient mice were challenged subcutaneously with the indicated doses of ovalbumin. 5 d after Ag challenge, cutaneous lymph nodes were collected and analyzed by flow cytometry for OT-II proliferation (bottom) and FoxP3 expression (top). (C and D) Spleen, mesenteric and cutaneous lymph nodes, small intestine lamina propria, and skin were collected from *Itgax*-Cre and *Itgax*-Cre *Irf4*^fl/fl^ mice. (C) Endogenous CD4^+^ T cell populations were analyzed by flow cytometry for FoxP3 expression. Numbers indicate percentage FoxP3^+^ within adjacent gate. Representative data from spleen and small intestine lamina propria are shown. (D) Cumulative data from several independent analyses are provided. Symbols represent individual mice. Errors bars represent mean ± SD. (E–H) CD45RB^hi^ CD4^+^ T cells were sorted from *Itgax*-Cre mice and transferred into RAG1^−/−^ recipients alone or with *Itgax*-Cre (Cont) CD45RB^lo^ CD4^+^ T cells or *Itgax*-Cre *Irf4*^fl/fl^ (cKO) CD45RB^lo^ CD4^+^ T cells. (E) Ear thickness measured by caliper at the completion of the study. (F and G) Pathology disease scoring for ear (F) and colon (G) tissue collected at the completion of the study. (E–G) Error bars represent mean ± SD. (H) Kaplan–Meyer curve for incidence of skin disease over the course of the study.

IRF4 has been shown to control DC priming of Th2 and Th17 T_eff_ responses in vivo (Gao et al., 2013; Schlitzer et al., 2013). However, as noted earlier, a role for IRF4 in programming DCs for induction of T_reg_ in vivo has not been explored. We tested this possibility by transferring dye-labeled ovalbumin-specific OT-II T cells into *Itgax*-Cre *Irf4*^fl/fl^ or *Itgax*-Cre control mice and subsequently challenging recipient mice by subcutaneous administration of low doses of cognate antigen. After antigenic challenge (5 d), we collected cutaneous lymph nodes for analysis. Consistent with our previous studies (Vander Lugt et al., 2014), we found reduced proliferation of MHCII-restricted OT-II T cells in *Itgax*-Cre *Irf4*^fl/fl^ mice (Fig. 4, A and B). Furthermore, *Itgax*-Cre *Irf4*^fl/fl^ mice showed impaired priming of FoxP3^+^ T_reg_ (Fig. 4, A and B). Importantly, reduced elicitation of T_reg_ was observed even when comparing antigen doses that allow for similar OT-II proliferation in *Itgax*-Cre *Irf4*^fl/fl^ mice and their control counterparts (3- and 1-µg doses, respectively). This suggests that IRF4 has a critical role in DC priming of peripheral T_reg_ induction, independent of its role in stimulating T cell proliferation, the latter via controlling efficient MHCII antigen presentation by DCs. Thus the in vivo findings, coupled with those obtained using our in vitro BMDC system, substantiate a critical role of IRF4 in programming tolerogenic T cell responses.

We next examined endogenous T_reg_ in *Itgax*-Cre *Irf4*^fl/fl^ mice. We found a small but reproducible decrease in FoxP3^+^ T cells in spleen and lymph nodes (Fig. 4, C and D). In contrast, we found a marked reduction in FoxP3^+^ T cells in peripheral tissues such as skin and small intestine (Fig. 4, C and D). These results are consistent with defective peripheral T_reg_ induction or homeostasis. Despite the reduced peripheral T_reg_ populations, we did not observe evidence of autoimmunity in *Itgax*-Cre *Irf4*^fl/fl^ mice at any age (unpublished data). This is likely a result of concomitant impairment of T_eff_ responses in *Itgax*-Cre *Irf4*^fl/fl^ mice. It should be noted that the *Itgax*-Cre strain used in our studies has been observed to be “leaky,” with Cre expression detected in CD11c-negative lineages, including up to 25% of T cells (Schlitzer et al., 2013). This could affect interpretation of the endogenous T_reg_ data. However, given the profound and selective reduction in peripheral T_regs_ compared with circulating T_regs_, our results nonetheless must reflect, at least in part, a DC-intrinsic role for IRF4 in programming regulatory function.

We next assessed the generation of functionally immunosuppressive peripheral T_reg_ in *Itgax*-Cre *Irf4*^fl/fl^ mice. We made use of a widely used model for peripheral autoimmunity wherein we transferred purified naive CD45RB^hi^ CD4^+^ T cells from *Itgax*-Cre control mice into T cell–deficient *Rag2*^−/−^ mice (Powrie et al., 1993). As expected, recipient mice developed skin autoimmunity and colitis, as measured by increased ear thickness and pathological scoring of skin and intestinal inflammation (Fig. 4, E–H). Cotransfer of CD45RB^lo^ antigen-experienced CD4^+^ T cells, which included T_regs_, from *Itgax*-Cre mice prevented the development of disease (Fig. 4, E–H). In contrast, transfer of CD45RB^lo^ CD4^+^ T cells from *Itgax*-Cre *Irf4*^fl/fl^ mice failed to suppress disease. This was despite transfer of higher numbers of CD45RB^lo^ CD4^+^ T cells from *Itgax*-Cre *Irf4*^fl/fl^ mice relative to their control counterparts to ensure delivery of equivalent numbers of FoxP3^+^ T_regs_. Collectively, these results demonstrate a critical and novel role for IRF4 not only in DC priming of peripheral T_reg_ induction, but also in imparting efficient immunosuppressive capacity to such cells.

### In vitro model for functionally divergent DC maturation programs

**Figure 5.**
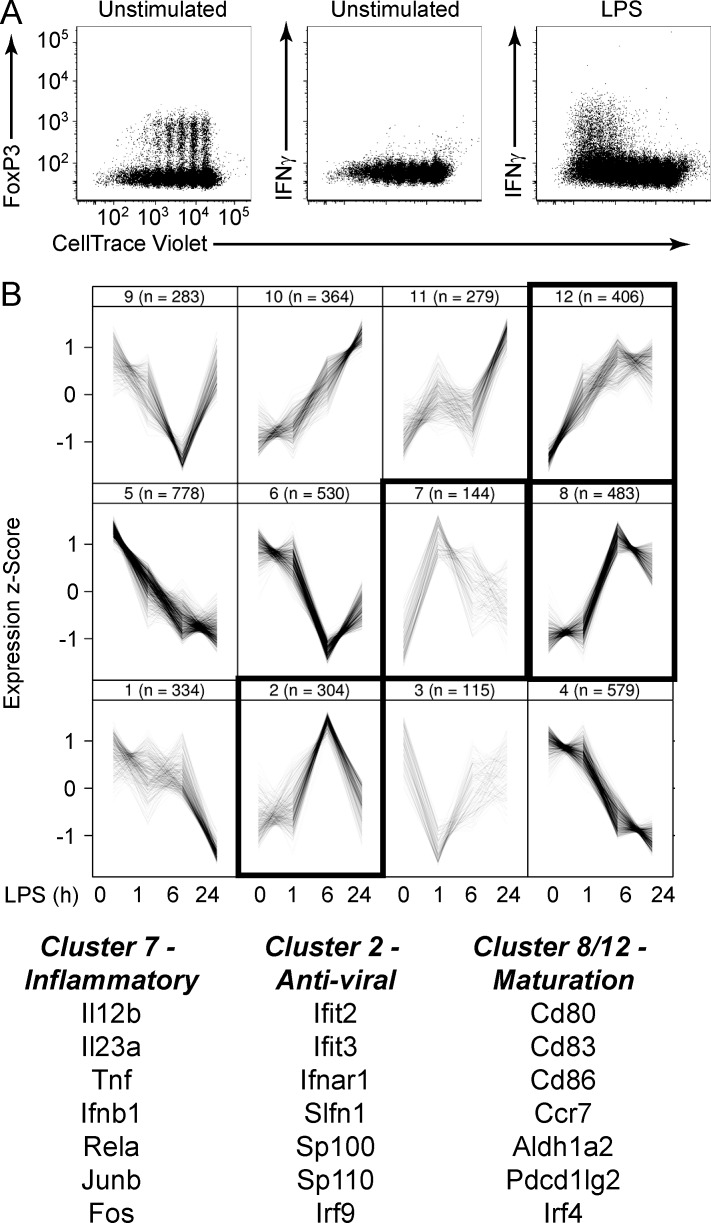
**In vitro model for functionally divergent maturation programs.** (A) Ovalbumin-loaded BMDCs were left unstimulated or stimulated with LPS and then co-cultured with CellTrace Violet–labeled naive OT-II T cells for 5 d. T cells were subsequently analyzed by flow cytometry for dye dilution and expression of FoxP3 and IFNγ. Data are representative of more than three experiments. (B) BMDCs were stimulated with LPS for 0, 1, 6, and 24 h, and microarray gene expression analysis was performed. Data were compiled for three independent experiments. LPS-regulated genes were organized into 12 clusters according to similar kinetic profiles. Cluster 7 showed a strong inflammatory signature, whereas cluster 2 showed a strong antiviral signature. Clusters 8 and 12 included genes encoding costimulatory and regulatory signals. Representative genes of interest within each module are annotated. Numbers in parentheses indicate number of genes assigned to that module. Traces within each box represent mean expression of individual genes averaged over three experiments at the indicated time points after stimulation.

We next extended our in vitro system to analyze the regulatory basis of danger-induced licensing of immunogenic function to contrast it with DC priming of T_reg_ differentiation. To accomplish this, we stimulated ovalbumin-pulsed BMDCs with LPS and assayed for their ability to prime T_eff_ differentiation of naive OT-II T cells. As demonstrated earlier, BMDCs undergoing spontaneous (steady-state) maturation promoted naive T cell differentiation into T_reg_ (Fig. 5 A, left). Notably, they did not promote T_eff_ differentiation, as assessed by IFNγ production (Fig. 5 A, middle). In striking contrast, LPS signaling programmed ovalbumin-pulsed BMDCs to efficiently promote T_eff_ differentiation (Fig. 5 A, right). Thus, the BMDC model system manifests two fundamental and distinctive features of DC maturation and programming: steady-state maturation is associated with T_reg_-inducing potential, and danger signal–induced maturation is coupled with immunogenic licensing.

To gain insight into the molecular mechanisms underlying the divergent functional states of phenotypically mature DCs, we analyzed the BMDC transcriptional response associated with LPS-stimulated immunogenic licensing. We performed microarray-based gene expression analysis of BMDCs stimulated with LPS for 1, 6, and 24 h and found that 4,603 genes were differentially expressed at one or more time points (Fig. 5 B). These genes exhibited complex patterns of expression but could be organized into distinct kinetic clusters, consistent with previous studies (Amit et al., 2009).

We noted two particularly interesting gene clusters that displayed transient expression with either early (2 h, cluster 7) or delayed (6 h, cluster 2) induction (Fig. 5 B). These clusters were enriched for inflammatory cytokine and antiviral response genes, respectively. The expression kinetics of these gene modules were consistent with well-characterized inflammatory and IFN-driven signaling axes associated with immune activation. In contrast, genes associated with costimulation, migration, and tolerogenic signals did not display such transient patterns and segregated within separate kinetic clusters (clusters 8 and 12). These distinctive patterns of gene expression supported the idea that a core DC maturation program is integrated with “accessory” functional programs associated with the induction of either tolerance or immunity.

### Distinct regulatory determinants predicted for divergent DC states

**Figure 6.**
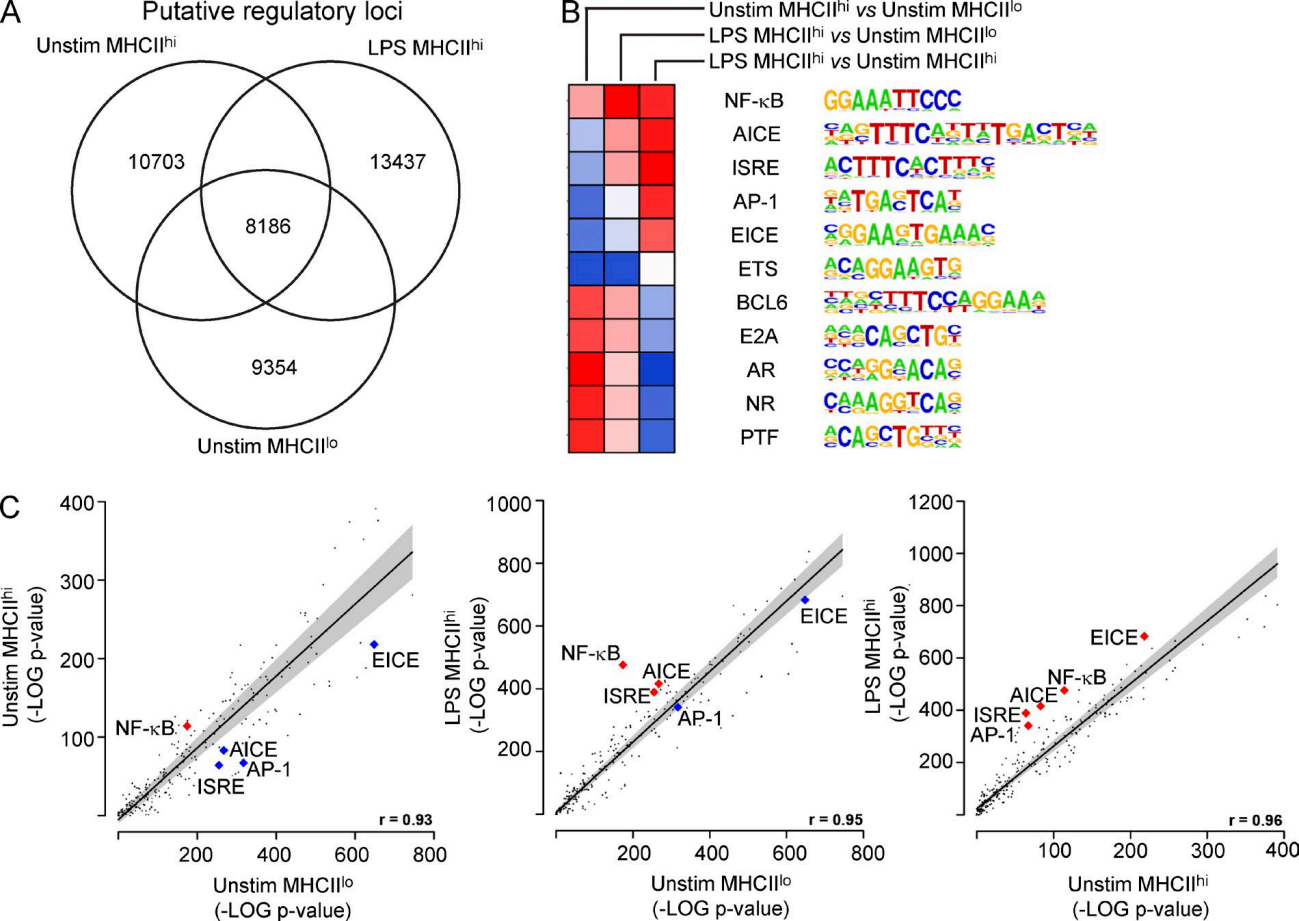
**Distinct regulatory determinants predicted for divergent functional states.** (A) ChIPseq analysis of genome-wide histone H3K27 acetylation was used to identify putative regulatory regions in unstimulated (Unstim) MHCII^lo^, unstimulated MHCII^hi^, and LPS-stimulated MHCII^hi^ BMDCs. Numbers of unique and common sequences are indicated by Venn diagram. (B and C) Genomic sequences associated with putative regulatory regions uniquely associated with each state were analyzed for the presence of motifs recognized by TFs compiled in the HOMER database. Differential enrichment of select motifs is displayed as a heatmap. Motifs are represented in LOGO format. (C) Negative log_10_-transformed p-values for motif enrichment in MHCII^lo^ versus unstimulated MHCII^hi^ (left), MHCII^lo^ versus LPS-stimulated MHCII^hi^ (middle), and unstimulated MHCII^hi^ vs. LPS-stimulated MHCII^hi^ (right) cells are represented as scatter plots. Red and gray lines indicated lines of linear regression and 90% confidence interval, respectively. *R* values for fit to regression lines are indicated at bottom right. Data points for NF-κB, AP-1, ISRE, Ets-IRF composite element (EICE), and AP-1-IRF composite element (AICE) motifs are annotated.

The differential gene expression patterns associated with steady-state and LPS-induced DC maturation suggested that they reflect alternative states of differentiation that are specified by distinctive regulatory determinants. To explore this possibility, we profiled the chromatin landscape of purified unstimulated MHCII^lo^ immature, unstimulated MHCII^hi^ steady-state mature, and LPS-stimulated MHCII^hi^ mature BMDCs. ChIPseq was performed for histone H3K27 acetylation and H3K4 trimethylation, as their focal distribution is indicative of putative genomic regulatory elements (Fig. 3). Histone profiling revealed 8,186 putative regulatory regions that were common to all three states, as well as ∼10,000 regions that were unique for each state (Fig. 6 A).

We extracted the genomic sequences associated with both common and unique regions and searched for enrichment of known TF binding motifs. We reasoned that motifs enriched in one state versus another would implicate candidate TFs that function in specification of particular states. As shown in Fig. 6 (B and C), the Ets-IRF composite element was highly enriched within all regions, particularly within shared regions. Immune-specific members of the Ets (PU.1/SpiB) and IRF (IRF4/IRF8) TF families bind cooperatively to such motifs and regulate expression of genes involved in DC maturation and MHCII antigen presentation (Brass et al., 1999; Singh et al., 2013; Vander Lugt et al., 2014). This analysis validated the chromatin modification and computational analyses as a means of inferring regulatory factors that control DC maturation and function.

Motif analysis of regulatory regions unique to steady-state MHCII^hi^ or LPS-stimulated MHCII^hi^ mature DCs relative to those in MHCII^lo^ cells revealed several interesting patterns (Fig. 6 B). Steady-state mature DCs showed an enrichment of motifs targeted by Bcl6, nuclear receptors, and E2A family factors and depletion of motifs targeted by AP-1 and IRFs. In contrast, LPS mature DCs exhibited an enrichment of motifs targeted by nuclear factor (NF)-κB factors and the prototypic IRFs as well as AP-1–IRF complexes. The latter finding was consistent with the well-established role of NF-κB and IRFs in mediating the inflammatory transcriptional response, including the gene sets associated with LPS-licensed immunogenicity (Fig. 5 B). This observation further validated our overall computational approach. Thus the identification of TF motifs that were differentially enriched between tolerogenic and immunogenic states provided evidence for distinct transcriptional determinants that program the functionally divergent DC states.

### NF-κB programming of immunoregulatory potential

Our analyses thus far had suggested that DC immunogenic programming is regulated by transcriptional determinants that are distinct from those that control the core maturation as well as tolerogenic programs. To directly test this hypothesis, we sought to perturb the NF-κB system, as it was implicated by chromatin analysis as a selective determinant of immunogenic programming. Notably, the NF-κB motif was the most highly enriched within putative regulatory genomic regions unique to the LPS-stimulated mature DC state, as illustrated by its degree of displacement from the line of linear regression in Fig. 6 C.

**Figure 7.**
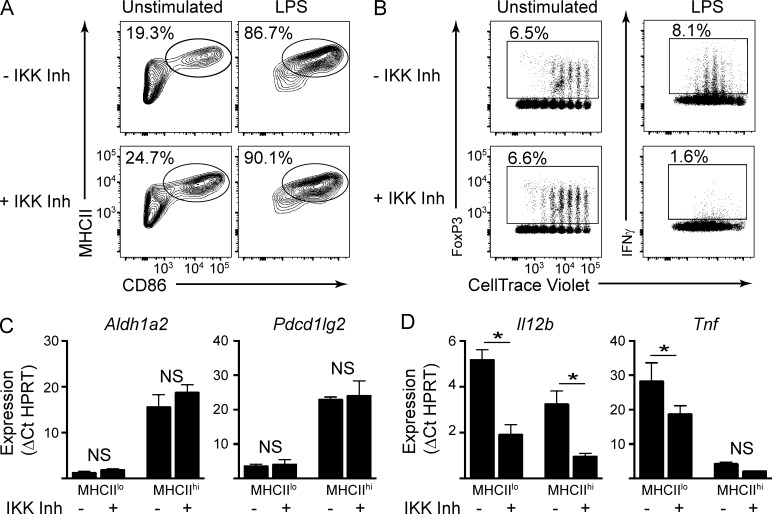
**Distinctive programming for tolerogenic maturation and immunogenicity.** (A) BMDCs were generated in the presence or absence of IKK inhibitor (Inh) and then left unstimulated or stimulated overnight with LPS. CD11c^+^ cells were analyzed by flow cytometry for expression of MHCII and CD86. Data are representative of more than three independent experiments. (B) BMDCs were generated in the presence or absence of IKK inhibitor, loaded with ovalbumin, and left unstimulated or stimulated with LPS. MHCII^hi^ populations were purified and cultured with CellTrace Violet–labeled naive OT-II T cells for 5 d. T cells were analyzed by flow cytometry for dye dilution and expression of FoxP3 and IFNγ. Numbers indicate percentage within the adjacent gate. Data are representative of three independent experiments. (C) MHCII^lo^ and MHCII^hi^ populations were purified from unstimulated BMDCs cultured in the presence or absence of IKK inhibitor. Expression of the indicated genes was determined by Taqman RT-PCR. Bars indicate mean of three independent experiments ± SEM. (D) BMDCs purified as in C were stimulated for 6 h with LPS and assayed for the expression of the indicated genes. Bars indicate mean of three independent experiments ± SEM.

To confirm the role of NF-κB in specifically controlling the danger-induced components of maturation, we used a selective inhibitor of IκB kinase (IKK) as a means of acutely impairing activation of NF-κB factors. After performing titration analysis to determine optimal inhibitory concentrations (Fig. S3, A and B), we examined the effects of two chemically distinct IKK inhibitors on BMDCs. Importantly, IKK inhibition had no effect on the up-regulation of MHCII or costimulatory molecules, either in the steady-state or in response to LPS stimulation (Fig. 7 A).

We next examined the effects of IKK inhibition on DC function. As illustrated in Fig. 7 B, IKK inhibitor–treated MHCII^hi^ cells purified from unstimulated BMDCs promoted T_reg_ differentiation to levels comparable with their untreated counterparts. We further verified that T_reg_ generated under these conditions were suppressive in functional assays (Fig. S5). In striking contrast, IKK inhibitor–treated, LPS-stimulated BMDCs were diminished in their ability to direct T_eff_ differentiation (Fig. 7 B, right). These results clearly demonstrate that the tolerogenic DC maturation program can be dissociated from the immunogenic program; the latter is dependent on activation of the NF-κB system.

We next determined whether IKK inhibition differentially impacted the expression of key genes identified earlier that are characteristic of either tolerogenic or immunogenic maturation. For this purpose, MHCII^lo^ immature and MHCII^hi^ steady-state mature populations were sorted from unstimulated BMDCs generated in the presence or absence of the IKK inhibitor. As shown in Fig. 7 C (and Fig. S4 A), the expression of *Aldh1a2*, *Pdcd1lg2*, *Cd83*, and *Ccl22* was unaffected by IKK inhibition. We also examined another important aspect of steady-state MigDC function, the ability of the cells to migrate in response to chemokine signals. We found that IKK inhibition had no effect on the expression of the CCR7 chemokine receptor, which is required for DC migration from tissues to lymph nodes, or on the ability of BMDCs to migrate in response to CCL19/CCL21 ligands (Fig. S4, E and F). Thus, IKK inhibition does not impact expression of key components of the steady-state tolerogenic maturation program.

Next, the sorted BMDC populations were treated with LPS in the presence or absence of IKK inhibitors and examined for expression of immunogenic components. IKK inhibition strongly impaired LPS-induced expression of cytokines such as IL-12, IL-23, TNF, and IL-6, at both transcript and protein levels (Figs. 7 D and S4, B and D). We note that the half-maximal inhibitory concentration (IC_50_) values of ∼60 nM for inhibition of cytokine gene expression were in close agreement with published IC_50_ values for the inhibition of IKKβ enzymatic activity by the IKK inhibitor (Waelchli et al., 2006). As expected, the LPS-induced degradation of IκB was impaired in the presence of inhibitor at the aforementioned concentrations (Fig. S3 C). Thus, IKK inhibition impairs expression of immunogenic cytokines and the priming of T_eff_ responses.

Interestingly, MHCII^hi^ steady-state mature DCs express much lower levels of most inflammatory cytokines in response to LPS compared with their MHCII^lo^ immature counterparts (Figs. 7 D and S4 B). This suggests that steady-state maturation might be accompanied by repression of the danger-induced program. Such a refractory state could be achieved by histone deacetylase–dependent silencing of inflammatory genes, as previously reported for macrophages (Foster et al., 2007). Thus, the steady-state and inflammatory maturation programs may cross-regulate one another through a mutual repression module.

Consistent with the idea of antagonistic programming, expression of the inflammatory cytokines IL-12 and TNF was slightly elevated in IRF4-deficient DCs (Fig. 2, D and F). Our in vitro results of opposing roles for IRF4 in regulating DC maturation and expression of polarizing cytokines are consistent with recent in vivo analysis (Akbari et al., 2014). This additionally validates the utility of our model system. Collectively, we demonstrate that the programming of the tolerogenic state in DCs that accompanies steady-state maturation is dependent on transcriptional determinants that are distinct from and appear to antagonize those required for immunogenic programming.

## Discussion

Environmental danger cues trigger DC maturation and stimulate DCs for enhanced antigen presentation and production of polarizing cytokines, which drive T cells to acquire effector function. The coincidence of DC maturation and immunogenic function in this context has been interpreted to signify that DC maturation and functional status are mechanistically linked binary states, i.e., immature DCs are tolerogenic whereas mature DCs are immunogenic. However, this simple model does not sufficiently capture the spectrum of DC function in tolerance or immunity. Steady-state MigDCs that promote tolerance express high levels of MHCII and costimulatory molecules and do not provoke autoimmune responses (Ruedl et al., 2000). Furthermore, different classes of pathogens can elicit distinctive transcriptional responses in DCs that lead to priming of varied T_eff_ states (Huang et al., 2001; Garber et al., 2012) . This suggests that distinct cues can stimulate DCs to adopt divergent mature states specialized to promote tolerance or immune responses, the latter tailored to the pathogen encountered. We explored the gene regulatory basis of these observations using an in vitro system that enables DC maturation and the priming of regulatory or effector T cell responses. In so doing, we provide evidence of a danger-independent maturation state with potential to promote T_reg_ differentiation.

We propose that a core program for maturation controls the up-regulation of antigen presentation and costimulatory functions to enable efficient engagement with antigen-specific T cells. We further propose that distinctive networks of genes and their unique underlying regulatory determinants are differentially engaged by either innocuous or danger signals to specify divergent DC functional properties. Importantly, our data indicate that tolerogenic functions can be up-regulated in a manner similar to immunogenic functions during DC maturation. Such modular programming enables a spectrum of DC activation states and an exquisite capability to shape tolerogenic or immunogenic responses.

Given that our conclusions have been derived from an in vitro system, their physiological relevance and possible caveats must be considered. Our system does not reproduce all distinctive features of tissue-resident DCs, which exist as heterogeneous subsets. Nonetheless, the system recapitulates key properties exclusively associated with peripheral DCs that have been genetically and functionally associated with tolerance regardless of DC subset, e.g., high expression of antigen presentation genes as well as PD-L2 and RALDH. We note that the presence of DCs in peripheral tissues that appear phenotypically mature and that have a critical role in peripheral tolerance is fairly well established. Our data provide a gene regulatory framework that not only helps to reconcile these observations with previous models of DC maturation and function but also begins to illuminate the molecular underpinnings.

Although we propose distinctive modes of regulation, we do not believe that maturation coupled with the acquisition of tolerogenic or immunogenic functions represent entirely independent programs. The diminished expression of danger-induced inflammatory signals in the context of steady-state maturation suggests that the tolerogenic program antagonizes these genes. Conceivably, the self-antigen presentation capabilities of steady-state mature DCs present a potential risk to the host should the immunogenic program become inappropriately activated. The steady-state maturation program may have evolved additional counterregulatory mechanisms to prevent this scenario. We also emphasize that our data do not address the nature of tolerogenic signals that direct the core maturation program. The identification of a large gene set uniquely expressed during steady-state BMDC maturation, as well as the identification of TF motifs enriched in associated genomic regulatory regions, suggests a steady-state tolerogenic program that is controlled by distinct signals and transcriptional determinants.

We have focused on IRF4 and NF-κB together to illustrate the distinctive regulation of tolerogenic versus immunogenic maturation and function. The control of diverse functional programs in DCs clearly requires greater regulatory complexity than is captured by our reductionist analysis. IRF4, for example, has been shown to have key roles in regulating immunogenic DC functions (Gao et al., 2013; Schlitzer et al., 2013; Williams et al., 2013). Our ChIP-seq analysis shows that IRF4 targets genes encoding tolerogenic signals as well as inflammatory genes. In this regard, it will also be important to consider divergent roles for IRF4 and the closely related IRF8. IRF4 and IRF8 are reciprocally expressed among DC subsets (Singh et al., 2013). We note that ALDH and PD-L2 are expressed in tolerogenic IRF8-expressing MHCII^hi^ CD103^+^ CD11b^−^ DCs in the gut (Fig. S2, A and B) and that these molecules continue to be expressed in IRF8-dependent DCs in *Itgax*-Cre *Irf4*^fl/fl^ mice (unpublished data). This suggests that IRF8 may be functionally interchangeable with IRF4 in regulation of these genes. IRF8 has a well-established role in promoting the expression of the p40 subunit of key immunogenic signals IL-12 and IL-23 (Giese et al., 1997), whereas IRF4 has been proposed to selectively regulate IL-33 and the p19 subunit of IL-23 (Schlitzer et al., 2013; Williams et al., 2013). One could imagine that IRF4- and IRF8-expressing DC subsets might exhibit somewhat different capabilities in how they shape the skewing of MHCI- and MHCII-restricted immune responses. The molecular mechanisms by which IRF4 regulates opposing T_eff_ and T_reg_ priming potential in DCs undergoing danger-induced or steady-state maturation, respectively, remain to be established.

The regulatory role of NF-κB factors is also likely to involve greater complexity than addressed in our studies. A recent study of mice harboring DCs genetically deficient in IKKβ suggested a role for NF-κB in homeostatic functions of DCs (Baratin et al., 2015). In contrast, our analysis with IKK inhibitors suggested that robust NF-κB activation was dispensable for canonical features of steady-state DC maturation. We note that some residual NF-κB activity likely remains at the inhibitor concentrations used in our studies. A role for low levels of tonic NF-κB signaling may therefore explain the discrepancy between these results. Alternatively, the difference may reflect the consequences of long-term inactivation of NF-κB signaling achieved by gene deletion as opposed to the transient inhibition of NF-κB activity that is mediated using a pharmacologic inhibitor. Future work will be required to address this and other fundamental questions regarding the regulatory programs orchestrating diverse states of DC maturation and function.

## Materials and methods

### Mice

*Itgax-*Cre (Caton et al., 2007), *Irf4*^fl/fl^ (Klein et al., 2006), OT-II (Barnden et al., 1998), and *Rag1*^−/−^ (Mombaerts et al., 1992) mice have been described. *Itgax*-Cre mice harbor a 160-kb BAC transgene of the *Itgax* locus encoding the Cre recombinase under the control of the *Itgax* promoter. *Irf4*^fl/fl^ mice were engineered with LoxP sites flanking exons 1 and 2 of *Irf4*. This strain was additionally engineered such that excision of the LoxP-flanked sequence results in juxtaposition of a promoter with eGFP, resulting in expression of eGFP in cells that have deleted IRF4. OT-II mice harbor a transgene encoding a T cell receptor that recognizes ovalbumin peptide presented in I-A^b^ MHCII molecules. *Rag1*^−/−^ mice were engineered with a Neomycin cassette knocked in to the *Rag1* locus to disrupt the NLS and zinc finger, resulting in a nonfunctional protein. Wild-type C56BL/6J and *Rag1*^−/−^ mice were obtained from the Jackson Laboratory. Experimental mice were cohoused in a specific pathogen–free barrier facility in accordance with Genentech LARC guidelines. Experiments were conducted with age- and sex-matched mice 6–12 wk of age. All animal study protocols were conducted in accordance with guidelines approved by the Institutional Animal Care and Use Committee at Genentech.

### Statistical analysis

Sample sizes were chosen empirically to ensure adequate statistical power and were in line with accepted standards for the techniques used. No samples from experiments were excluded from analysis. Our study did not include randomized samples/animals. All experiments were performed without blinding. Statistical significance of our results was determined using appropriate statistical tests selected according to the distribution of the data being analyzed. Details for statistical testing are provided in the figure legends. We observed limited variance within each group of data, and statistical analysis compared groups with similar variance.

### GM-CSF and IL-4 BMDC cultures

Bone marrow was collected by aspiration from mouse femurs and tibias and processed to generate single-cell suspensions. After red blood cell lysis, progenitors were enriched by negative selection by staining with biotinylated anti-CD4 (RM4-5; BD), anti-CD5 (53-7.3; BD), anti-CD8α (53-6.7; BD), anti-CD19 (1D3; BD), and anti-B220 (RA3-6B2; BD) followed by magnetic depletion with antibiotin microbeads (#130-090-485; Miltenyi Biotec). Enriched progenitors were plated at 5 × 10^5^ cells/well in 24-well plates and cultured in RPMI supplemented with 10% FCS, penicillin/streptomycin, 10 mM Hepes, 2-mercaptoethanol, 10 ng/ml recombinant murine GM-CSF (Peprotech), and 5 ng/ml rmIL-4 (Peprotech). Every other day, half the medium was removed and replaced with fresh differentiation medium. After 6 d of culture, repeated pipetting was used to collect loosely adherent cells for analysis.

### Enrichment of dendritic cells from lymphoid tissues

To facilitate analysis of rare DC populations, an enrichment step was performed before analysis. Mice were killed, and tissues aseptically removed. Spleen, cutaneous lymph nodes, or mesenteric lymph nodes from five mice were pooled and processed to single-cell suspensions. B and T cells were depleted using negative selection with biotinylated anti-CD19 (rat; clone 1D3; BD), anti-B220 (rat; clone RA3-6B2; BD), and anti-CD3 (hamster; clone 145-2C11; BD) followed by depletion with antibiotin microbeads according to the manufacturer’s protocol.

### Flow cytometry

Single-cell suspensions were washed in PBS containing 5 mM EDTA and 0.5% BSA. The following antibodies were used for analysis: anti-CD11c (hamster; clone N418; eBioscience); anti-MHCII [I-A] (rat; clone NIMR-4; eBioscience); anti-CD11b (rat; clone M1/70; eBioscience); anti-CD103 (hamster; clone 2E7; BioLegend); anti–PD-L2 (rat; clone TY25; eBioscience); anti-FoxP3 (rat; clone FJK-16s; eBioscience); anti-IFNγ (rat; clone XMG1.2; eBioscience); anti-Vβ5 (mouse; clone MR9-4; BD); and anti-CD86 (rat; clone GL-1; BioLegend). Staining for intracellular FoxP3 and IFNγ expression was performed using FoxP3/TF staining buffer kit (#00-5523; eBioscience). Staining for ALDH activity was performed as previously reported using Aldefluor reagent kit (#01700; STEMCELL Technologies). Flow cytometry data were analyzed using Flowjo software.

### In vitro antigen presentation assays

#### T cell labeling

Spleens were aseptically collected from OT-II mice and processed to generate single-cell suspensions. CD4^+^ CD62L^+^ naive OT-II T cells were isolated by magnetic beads according to the manufacturer’s instructions (#130-104-453; Miltenyi Biotec). T cells were fluorescently labeled with CellTrace Violet (#C34557; Molecular Probes) according to the manufacturer’s instruction.

#### T_reg_ assay

BMDCs were generated as described. Endotoxin-free ovalbumin (EndoGrade ova, # 321001; Hyglos) was added to culture medium on day 4 and left in medium overnight. Day 5 Ag-loaded, CD11c^+^ cells were purified by positive selection with magnetic microbeads (#130–052-001; Miltenyi Biotec). Alternatively, CD11c^+^ MHCII^lo^ or CD11c^+^ MHCII^hi^ cells were purified by cell sorting. 10^5^ OT-II T cells (labeled with CellTrace Violet as detailed above) were added with 10^4^ purified BMDCs per well into a 96-well round-bottom tissue culture plate. After 5 d of co-culturing, cells were collected for flow cytometric analysis.

#### T_eff_ assay

BMDCs were generated and loaded with OVA antigen as detailed in the previous section. Day 5 BMDCs were stimulated overnight with 100 ng/ml LPS (L4391; Sigma-Aldrich) and purified on day 6. After 5 d of co-culturing, T cells were restimulated with Cell Stimulation Cocktail (#00-4970; eBioscience) for 6 h then collected for flow cytometric analysis.

### Public microarray gene expression data for migratory DC subsets

Microarray gene expression data for select DC subsets were downloaded from the ImmGen database (http://www.immgen.org; Heng and Painter, 2008). Data files were selected and categorized as follows: lymphoid tissue resident [DC_4+_SLN, DC_4+_Sp, DC_8+_SLN, DC_8-4-11b+_Sp, DC_8-4-11b-_Sp, DC_8+_Sp], peripheral tissue resident [DC_103+11b+_SI, DC_103+11b-_Lu, DC_103+11b-_Lv, DC_103+11b-_SI, DC_103-11b+24+_Lu, DC_103-11b+F4/80lo_Kd, DC_103-11b+_Lv], and migratory [DC_IIhilang-103-11blo_SLN, DC_8-4-11b+_MLN, DC_103-11b+_LuLN, DC_IIhilang-103-11b+_SLN, DC_8-4-11b+_SLN, DC_8-4-11b-_MLN, DC_8-4-11b-_SLN, DC_IIhilang+103+11blo_SLN, DC_103+11b-_LuLN]. To analyze gene expression data, we log-transformed normalized microarray intensity levels as provided in the original publications and databases. When necessary, we used median expression to summarize microarray probe-level data and used mean expression across biological replicates. For visualization purposes, gene expression data were Z-transformed as discussed in the figure legends. We performed genome-wide analysis for correlated expression of individual genes with *Aldh1a2* by calculating the Pearson correlation coefficients and significance. To account for multiple testing, we used the method of Benjamini and Hochberg and selected genes that had an adjusted p-value of ≤0.0005.

### BMDC microarray and RT-PCR gene expression analysis

#### Unstimulated MHCII^lo^ versus MHCII^hi^ BMDCs

Analysis was performed as previously reported (Vander Lugt et al., 2014). In brief, BMDCs cultured for 6 d were stained for CD11c and MHCII expression and sorted by flow cytometry. Total RNA was isolated from purified CD11c^+^ MHCII^lo^ or CD11c^+^ MHCII^hi^ BMDCs using RNeasy Plus Minikit (#74134; Qiagen). RNA was hybridized to Agilent WMG 4 × 44k arrays. Preprocessing, normalization, and statistical analyses of microarray data were performed using the R programming language and packages from the Bioconductor suite of tools. Intensity data from two-color microarray scans were preprocessed using the normal + exponential background correction model (Ritchie et al., 2007). Background-corrected intensity data were then normalized between arrays using quantiles normalization (Bolstad et al., 2003). Differential expression analysis was performed using the *limma* package (Smyth, 2004).

#### LPS-stimulated gene expression analysis

BMDCs were generated as described and then stimulated for 0, 1, 6, or 24 h with 100 ng/ml LPS before collection on d 6. Three replicates were analyzed for each time point. RNA was processed and hybridized as detailed earlier. To identify differential genes between *Itgax-*Cre and *Itgax-*Cre *Irf4*^fl/fl^, we selected genes that had a Benjamini–Hochberg adjusted p-value <0.05, regardless of fold change. To identify genes differentially expressed between MHC-II^hi^ and MHC-II^lo^ BMDCs, we performed the same preprocessing and normalization steps, followed by linear model analysis. In this case, we fitted a model that included the MHCII classification, selecting genes that had a Benjamini–Hochberg adjusted p-value <0.001 with a minimum 1.5-fold change. For RT-PCR analysis of select genes in complemented BMDCs, cDNA was prepared using SuperScript II reverse transcription kit (#11754-050; Invitrogen). RT-PCR was performed using premixed primer/Taqman probe master mixes for *Aldh1a2* (Mm00501306_m1; #4331182; Thermo Fisher Scientific); *Pdcd1lg2* (Mm00451734_m1; #4331182; Thermo Fisher Scientific); *Cd83* (Mm00486868_m1; #4331182; Thermo Fisher Scientific); *Ccl22* (Mm00436439_m1; #4331182; Thermo Fisher Scientific); *Ccr7* (Mm01301785_m1; #4331182; Thermo Fisher Scientific); *Il12b* (Mm00434174_m1; #4331182; Thermo Fisher Scientific); *Tnf* (Mm00443258_m1; #4331182; Thermo Fisher Scientific); *Il23a* (Mm01160011_g1; #4331182; Thermo Fisher Scientific); and *il6* (Mm00446190_m1; #4331182; Thermo Fisher Scientific). Transcript levels were normalized to housekeeping gene *Hprt1* (Mm01545399_m1; #4331182; Thermo Fisher Scientific).

### ChIPseq analyses and IRF4 target gene identification

ChIPseq for IRF4 in BMDCs was performed as previously described (Glasmacher et al., 2012). In brief, BMDCs cultured from IRF4-deficient or wild-type control progenitors were fixed for 15 min with 4% PFA in PBS before extraction of chromatin. Extracts were sonicated with a Covaris E220. Fragment sizes of 200–500 bp were confirmed by Bioanalyzer. IRF4-bound fragments were immunoprecipitated overnight at 4°C using a polyclonal anti-IRF4 (goat; clone M-17x; Santa Cruz Biotechnology, Inc.). Cross-linking was reversed, and libraries were prepared for sequencing by Illumina HiSeq. For epigenetic ChIPseq analyses, BMDCs were sorted as unstimulated MHCII^lo^, unstimulated MHCII^hi^, or LPS-stimulated MHCII^hi^ cells. ChIPseq was performed for H3K27Ac (ab4729; Abcam) and H3K4me3 (ab8580; Abcam). We used BAM-formatted H3K27Ac aligned ChIPseq read files (mm10) for peak calling with MACS v1.3.7.1 (Zhang et al., 2008) on Galaxy v1.01.

### Adoptive transfer T_reg_ induction assay

Thy1.1^+^ OT-II T cells were purified and labeled as described above. 5 × 10^6^ labeled OT-II T cells were transferred by tail vein injection into *Itgax*-Cre or *Itgax*-Cre *Irf4*^fl/fl^ mice. 1 d after T cell transfer, recipient mice were challenged by subcutaneous challenge near the shoulders with 0, 1, or 3 µg ovalbumin. 5 d after antigen challenge, cutaneous lymph nodes were collected and processed for flow cytometric analysis. Thy1.1^+^ T cells were gated and analyzed for dye dilution and expression of FoxP3.

### Endogenous T_reg_ analysis

Lymphoid tissue T_regs_ were analyzed by flow cytometry as described above.

#### Skin

Backs of mice were shaved, and a 5-cm^2^ section of dorsal skin was removed, minced with scalpel, and digested with 50 µg/ml Liberase TM (#05401127001; Roche) and 10 U/ml DNase I (#M0303; New England Biolabs) in RPMI + 5% FBS at 37°C with rotation for 1 h. Digestion solution was transferred to GentleMacs C tubes (#130-093-237; Miltenyi Biotec); single-cell suspensions were created and filtered through 100-µm cell strainers. Viable cells were analyzed by flow cytometry after live/dead stain gating.

#### Small intestine

Small intestine between secum and stomach was taken and flushed with PBS before removal of Peyer’s patches. Intestine was opened longitudinally and sectioned into 1-cm pieces. Intestinal pieces were washed in RPIM + 10 mM EDTA at 37°C with rotation for 10 min followed by vortexing. Washes were repeated three times to remove epithelial cells. Washed intestinal pieces were then digested in RPMI + 5% FBS at 37°C with rotation for 45 min. Digestion solution was filtered through 100-µm cell strainers. Viable cells were analyzed by flow cytometry after live/dead stain gating.

### Adoptive transfer peripheral autoimmunity model

CD4^+^ T cells were enriched from spleens of *Itgax*-Cre or *Itgax*-Cre *Irf4*^fl/fl^ mice using negative magnetic depletion kit (#130-104-454; Miltenyi Biotec). CD4^+^ CD45RB^hi^ CD44^lo^ naive and CD4^+^ CD45RB^lo^ CD44^hi^ antigen-experienced populations were purified by cell sorting. 2 × 10^6^ CD45RB^hi^ *Itgax*-Cre cells were transferred by tail vein injection into RAG1^−/−^ recipients alone or with 5 × 10^5^ CD45RB^lo^ *Itgax*-Cre cells or 6 × 10^5^ *Itgax*-Cre *Irf4*^fl/fl^ cells. A sample of transferred CD45RB^lo^ cells was analyzed by flow cytometry to confirm transfer of equivalent FoxP3^+^ cells. Recipient mice were monitored biweekly for weight loss and skin disease, as identified by hair loss, erythema, and scaling. Disease was particularly evidence on ears and muzzle. At 12 weeks, animals were euthanized. Ears and colon were removed and scored for inflammation by a pathologist.

### Differential motif enrichment analysis

H3K27 acetylation ChIPseq peak intervals were intersected using BEDOPS v2.4.2 (Neph et al., 2012) to identify common (overlapping) and unique (nonoverlapping) peaks between the unstimulated MHCII^lo^, unstimulated MHCII^hi^, and LPS-stimulated MHCII^hi^ BMDC experimental conditions. We scanned the peak sequences for motif enrichment using HOMER (Heinz et al., 2010) with all vertebrate motifs. Deming regression analysis of the –log p-values for motif enrichment was performed using the method comparison regression package in R v3.1.3. The normalized orthogonal regression residuals were ranked from highest to lowest, and the top 10% of motifs were included in the clustering. The motifs were then clustered using the Euclidean distance metric and mean linkage hierarchical clustering in Genesis v1.7.6.

### IKK inhibitor studies

Commercially available IKK inhibitor VII (#505378; EMD Millipore) and IKK inhibitor XII (#401491; EMD Millipore) were used for these studies. Inhibitors were initially tested by culturing BMDCs in the continuous presence of a dose ranging from 1 nM to 10 µM. Subsequent studies were performed using only IKK inhibitor VII at a final concentration of 100 nM.

### Western blots

BMDCs were cultured in varying concentrations of IKK inhibitor and left unstimulated or stimulated with 100 ng/ml LPS for 1 h. Cells were collected and lysed in RIPA buffer supplemented with complete protease inhibitor (#11836153001; Roche). 40 µg RIPA heat-denatured lysate per sample was run on 12% trig-glycine polyacrylamide gel and transferred onto PDVF membranes. Membranes were blocked in 5% powdered milk in Tris-buffered saline/Tween buffer and blotted using anti-IκB primary (clone E130; #ab32518; Abcam) at 1:5,000 and goat anti-rabbit HRP secondary (#sc-2004; Santa Cruz Biotechnology, Inc.) at 1:10,000. Signal was developed using SuperSignal ECL substrate (#37070; Thermo Fisher Scientific) and detected using Hyperfilm (#28-9068-35; GE Healthcare).

### T_reg_ suppression assay

BMDCs were cultured in the presence or absence of IKK inhibitor and loaded with ovalbumin overnight. Ovalbumin-loaded BMDCs were co-cultured with naive Thy1.1^+^ OT-II T cells as described above. After 5 d, T cells were collected, and CD25^+^ cells were purified by cell sorting. Naive polyclonal CD4^+^ T cells were isolated from wild-type C56BL6/J mice using a magnetic bead kit (Miltenyi Biotec) and labeled with CellTrace Violet as described above. Labeled B6 T cells were added to 96-well plates at 5 × 10^4^ cells per well in 100 µl RPMI. Purified in vitro generated T_reg_ were added at ratios of 1:16 to 1:1 in 100 µl RPMI. Anti-CD3 and anti-CD28 antibodies were added to a final concentration of 5 and 1 µg/ml, respectively. After 3 d, T cells were collected and Thy1.1^−^ T cell proliferation was analyzed by flow cytometry.

### Dendritic cell migration assay

MHCII^lo^ and MHCII^hi^ cells were purified from unstimulated BMDC cultures. 10^5^ purified cells were suspended in serum-free RPMI and placed into the upper chamber of a cell migration assay kit (#CBA-105; Cell Biolabs). Serum-free RPMI with or without 50 ng/ml rCCL19 and CCL21 (Peprotech) was placed into the bottom chamber, and migration was allowed to proceed for 3 h. Migrated cells were quantified according to the manufacturer’s instructions.

### Online supplemental material

Fig. S1 contains comparative genomic analyses of BMDCs generated in our hands, in vitro–derived DCs generated in an alternative in vitro system (Helft et al., 2015), and various DC and immune subsets extracted from mouse tissues analyzed directly ex vivo (ImmGen database). Fig. S2 contains flow cytometric analysis of DC subsets extracted from mouse tissues and analyzed directly ex vivo for the expression of key tolerogenic molecules (PD-L2 and ALDH). Fig. S3 contains dose–response characterization of IKK inhibitors used in our studies. Fig. S4 contains additional analysis of gene expression relevant to maturation and function, extending the data provided in Fig. 7. Fig. S5 contains suppression assay data for in vitro differentiated T_regs_.

## Supplementary Material

Supplemental Materials (PDF)
