# Supplementary material for: Transcriptional determinants of tolerogenic and immunogenic states during dendritic cell maturation
Source: J Cell Biol. 2017 Mar 6;216(3):779–92. doi: 10.1083/jcb.201512012 (PMC5350508; doi:10.1083/jcb.201512012)
Supplement: Supplemental Materials (PDF) [file JCB_201512012_sm.pdf]

Vander Lugt et al., https://doi.org/10.1083/jcb.201512012

THE JOURNAL OF CELL BIOLOGY

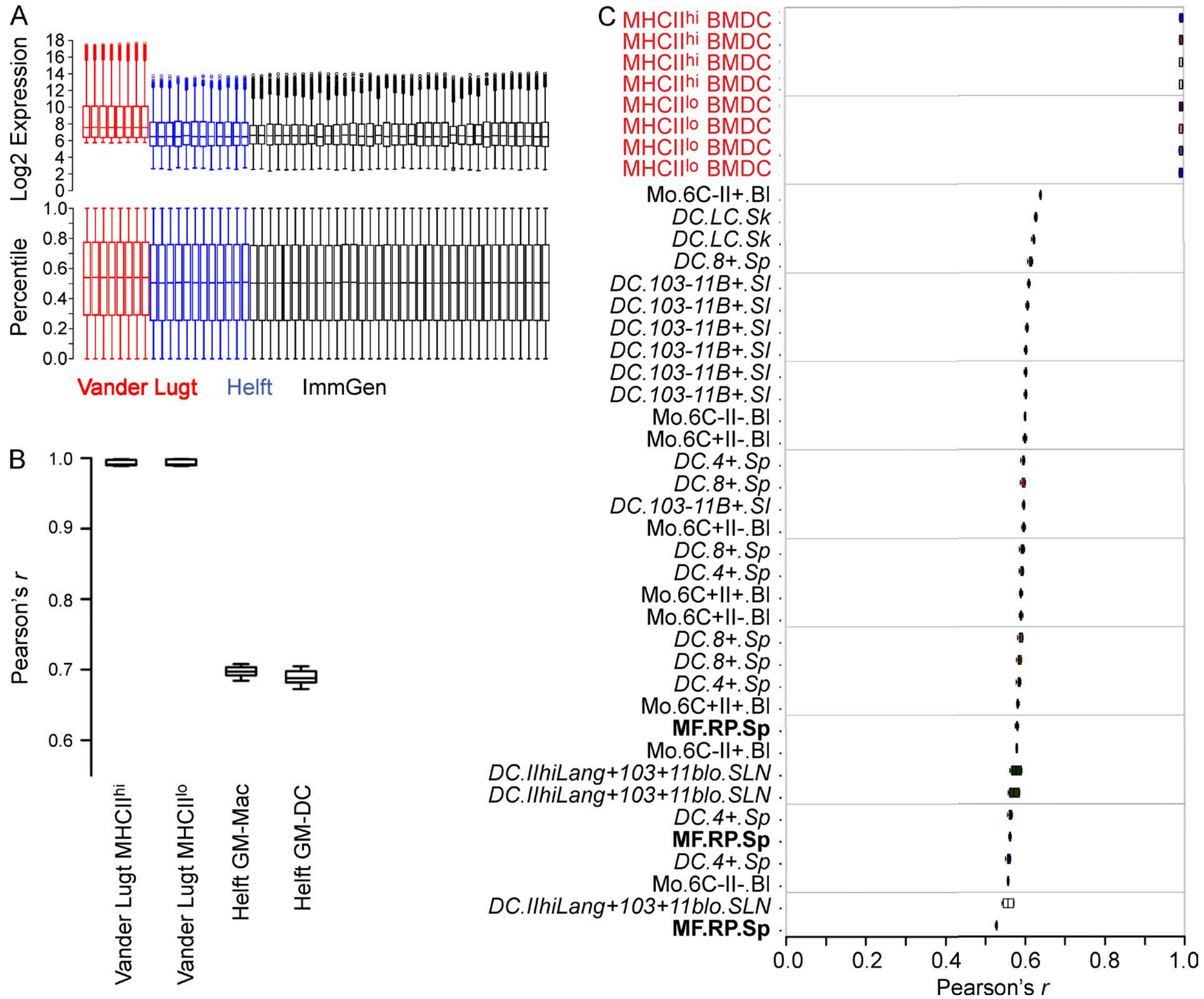

Figure S1. **GM-CSF and IL-4 BMDC system as a model for migratory DCs.** (A, top) Gene probeset log_2-transformed fluorescence intensity values from Agilent microarray profiles of GM-CSF/IL-4 BMDCs (Vander Lugt), Affymetrix microarray profiles of GM-CSF BMDCs (Helft), and Affymetrix microarray profiles of various populations of tissue DCs analyzed directly ex vivo (ImmGen) are represented as box-and-whisker plots. (bottom) Gene expression values were transformed by percentile ranking to normalize microarray platform fluorescence intensity biases in top panel. Boxes represent second and third quartiles of probe set signal intensities, and whiskers extend to maximum and minimum signals.(B) Percentile-transformed gene expression profiles from A were compared between GM-CSF/IL-4 MHCII^{hi} (n = 4), GM-CSF/IL-4 MHCII^{lo} (n = 4), GM-CSF CD115^{hi} (GM-Mac; n = 3), and GM-CSF CD135^{hi} (GM-DC; n = 3). Pearson's correlation coefficients are represented as box-and-whisker plots. (C) Analysis as in B comparing gene profiles between in vitro GM-CSF/IL-4 BMDC and ex vivo populations from the ImmGen database. Pearson's correlation coefficients are displayed in order of similarity. (B and C) Box-and-whiskers represent mean ± SD for correlation coefficients.

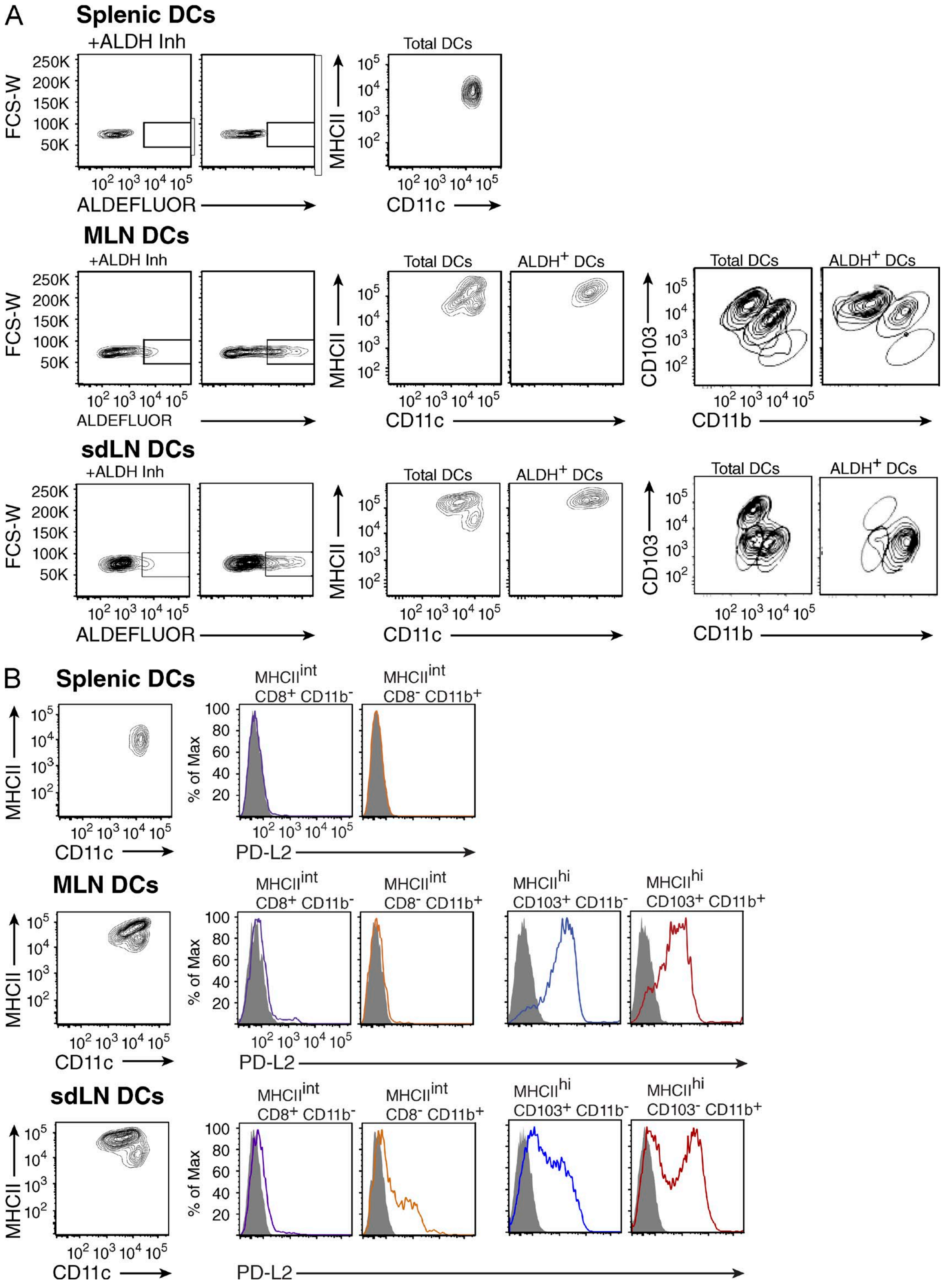

Figure S2. **Coordinated, restricted expression of regulatory signals in MigDCs.** (A) DCs were enriched from spleen, mesenteric lymph nodes (MLN), and skin-draining lymph nodes (sdLN). Fluorescently labeled ALDH substrate (ALDEFLUOR) was administered to isolated DCs in the presence or absence of ALDH inhibitor (Inh). Total and ALDH+ DCs were analyzed by flow cytometry for CD11c and MHCII. Data are representative of three independent experiments. (B) PD-L2 expression was analyzed by flow cytometry on DCs enriched as in A. MHCII^{int} and MHCII^{hi} subpopulations were distinguished by their expression of CD8, CD11b, and CD103 as indicated and separately analyzed. Filled gray histogram represents staining with isotype-matched control antibody. Data are representative of three independent experiments.

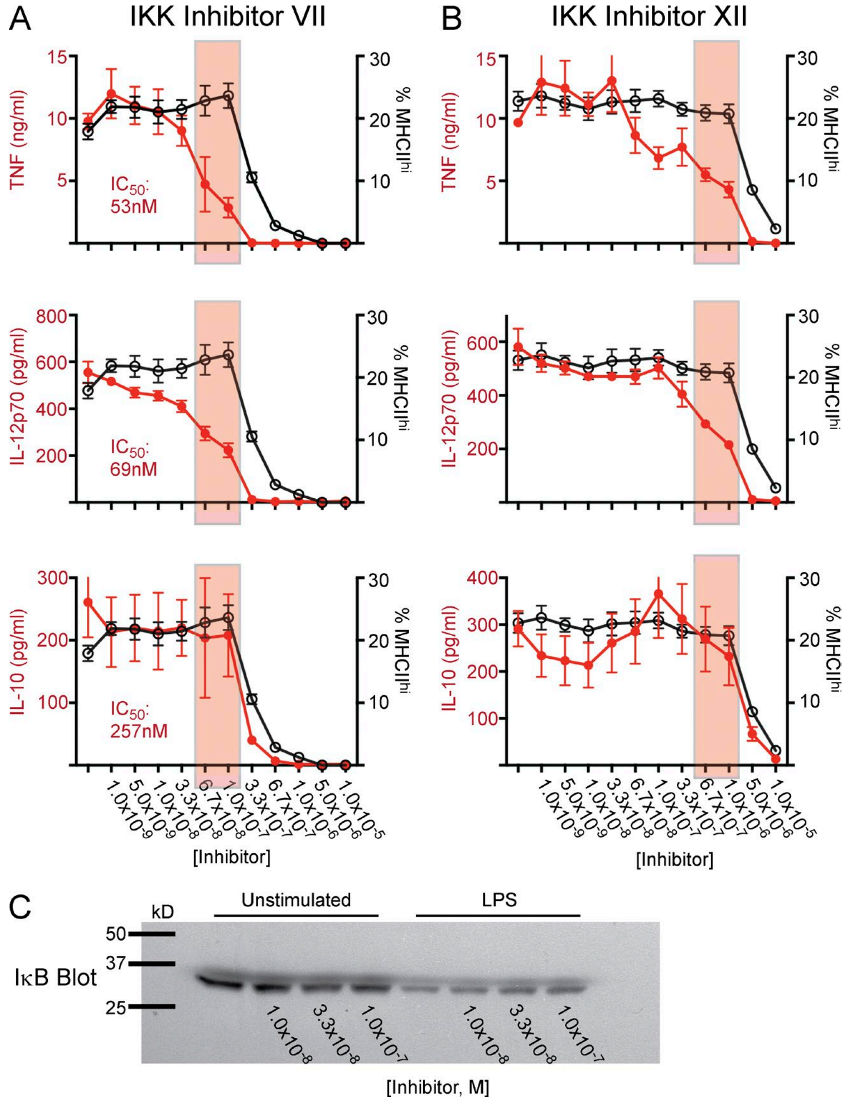

Figure S3. **IKK inhibitor dose optimization.** (A) BMDCs were generated in the presence of 1 pM to 10 μM IKK inhibitor VII (Calbiochem). The proportion of MHCII^{hi} cells in unstimulated BMDCs was determined by flow cytometry and is plotted in black using the right *y*-axis. Cultured BMDCs were stimulated for 6 h with LPS, and the concentration of TNF (top), IL-12p70 (middle), and IL-10 (bottom) in supernatants was determined by Luminex assay. Cytokine concentrations are displayed in red and plotted using the left *y*-axis. Symbols and bars represent mean ± SEM from three independent experiments. (B) Analysis as in A using IKK inhibitor XII (Calbiochem). Symbols and bars represent mean ± SEM from three independent experiments. (C) BMDCs were cultured in the indicated concentrations of IKK inhibitor VII and stimulated for 1 h with LPS. Cells were lysed in RIPA buffer, and lysates were analyzed by Western blot for IκB. Reference protein size ladder is annotated at left.

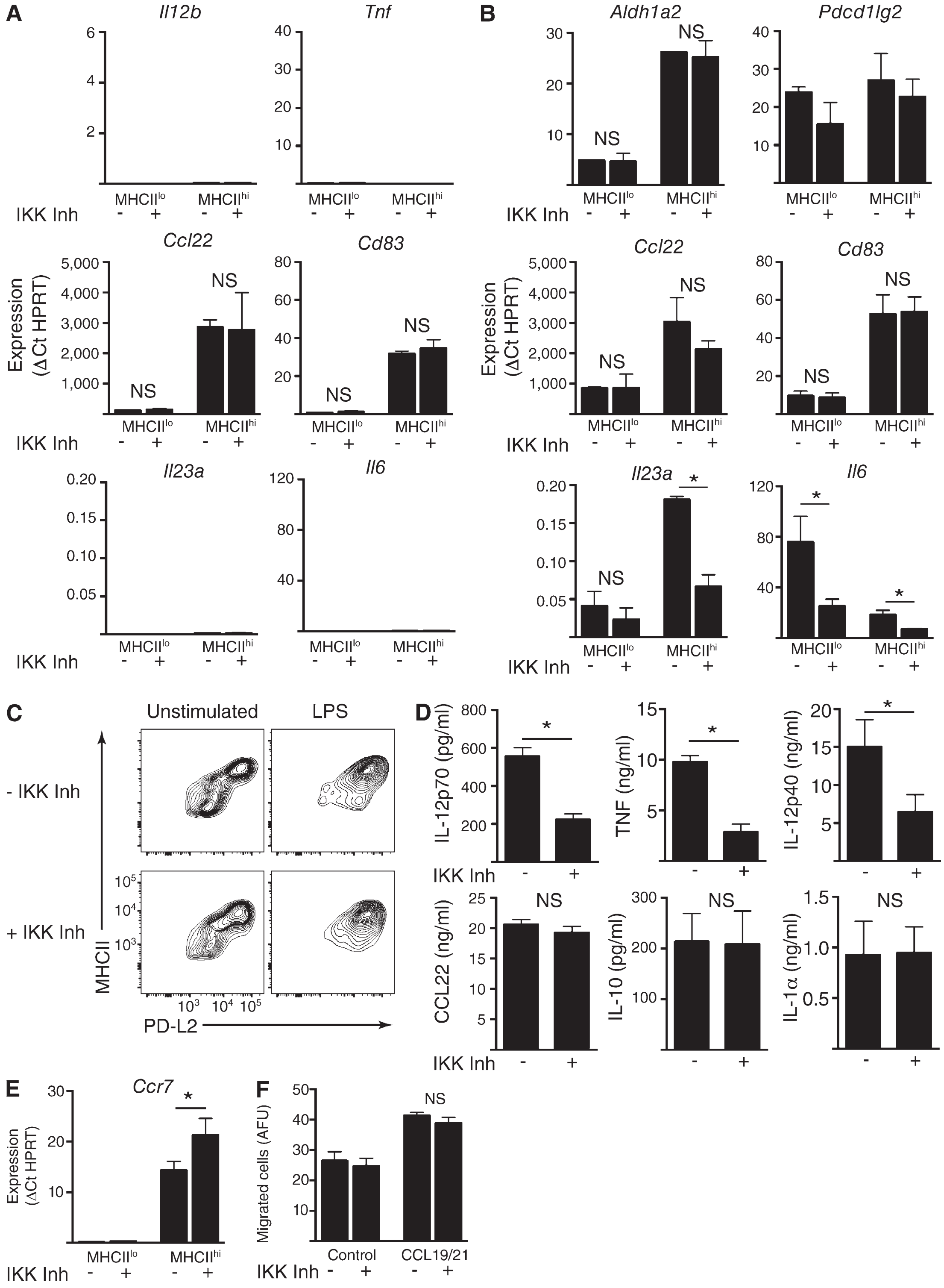

Figure S4. **Distinctive programming for tolerogenic maturation and immunogenicity.** (A) $MHCII^{lo}$ and $MHCII^{hi}$ populations were purified from unstimulated BMDCs cultured in the presence or absence of IKK inhibitor. Expression of the indicated genes was determined by Taqman RT-PCR. Bars indicate mean of three independent experiments ± SEM. (B) BMDCs purified as in A were stimulated for 6 h with LPS and then assayed for the expression of the indicated genes. (C) BMDCs were generated in the presence of absence of IKK inhibitor (Inh) and left unstimulated or stimulated overnight with LPS. $CD11c^{+}$ cells were analyzed by flow cytometry for expression of MHCII and PD-L2. Data are representative of three independent experiments. (D) BMDCs were generated in the presence or absence of IKK inhibitor and stimulated for 6 h with LPS. The concentration the indicated cytokines in supernatants was determined by Luminex assay. Bars indicate mean of three independent experiments ± SEM. (E) $MHCII^{lo}$ and $MHCII^{hi}$ populations were purified from unstimulated BMDCs cultured in the presence or absence of IKK inhibitor. Expression of *Ccr7* was determined by RT-PCR. Bars indicate mean of three independent experiments ± SEM. (F) $MHCII^{hi}$ cells were purified from BMDCs cultured in the presence or absence of IKK inhibitor and then analyzed in a Transwell migration assay either with no ligands or with CCL19 and CCL21. Cells migrating through the Transwell membrane were quantified using a fluorometric assay. Bars represent data in arbitrary fluorescence units (AFU) from three independent experiments ± SEM. Where indicated, statistical significance was assessed by two-tailed *t* test.

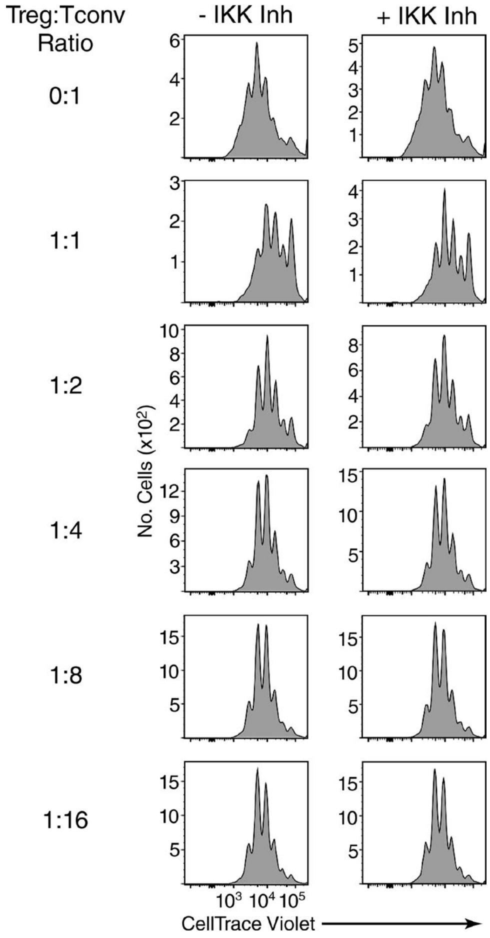

Figure S5. **IKK inhibitor–treated DCs induce functional T_{reg}.** BMDCs were generated in the presence or absence of IKK inhibitor and loaded with ovalbumin. $MHCII^{hi}$ populations were purified and cultured with CellTrace Violet–labeled naive Thy1.1^{+} OT-II T cells for 5 d. CD25^{+}Vβ5^{+} cells (T_{reg}) were sorted, and their suppressor activity was determined by co-culturing with polyclonal CellTrace Violet–labeled CD4^{+} T cells (Tconv) at the indicated ratios. T cell proliferation was induced with soluble αCD3 and αCD28. After 3 d, T cells were collected, and Thy1.1^{-} Tconv were analyzed by flow cytometry for dye dilution. Data are representative of three independent experiments.
